# 한국 성인 여성의 건강정보이해능력 및 관련 요인: 2023 국민건강영양조사 자료의 상관성 연구

**DOI:** 10.4069/whn.2026.01.14

**Published:** 2026-03-31

**Authors:** Mijong Kim, Hyunju Chae

**Affiliations:** 1 Department of Nursing, Hannam University, Daejeon, Korea; 1 한남대학교 간호학과; 2 Department of Nursing, Sangmyung University, Cheonan, Korea; 2 상명대학교 간호학과

**Keywords:** Adult, Health behavior, Health literacy, Women

## Abstract

**Purpose:**

This study examined health literacy and identified factors associated with health literacy among adult women.

**Methods:**

This correlational study was based on a secondary analysis of data from the 2023 Korea National Health and Nutrition Examination Survey (KNHANES). The study sample included 3,333 women aged 19 years or older. Data were analyzed using complex sample design procedures in SPSS ver. 20.1.

**Results:**

The mean health literacy score was 30.25 out of 40, indicating moderate health literacy. Scores were relatively low for items assessing the ability to evaluate the reliability of health information obtained from the internet or media and to use such information for health-related decision-making. Overall, 32.2% of adult women demonstrated an excellent level of health literacy. Lower education levels (≤elementary school: B=−4.31, *p*<.001; middle school: B=−2.17, *p*<.001; high school: B=−0.84, *p*<.001), longer weekday sleep duration (≥9 hours: B=−1.54, *p*=.003), poorer perceived health (bad: B=−1.49, *p*<.001; ordinary: B=−0.82, *p*<.001), older age (≥65 years: B=−1.43, *p*=.001; 45–64 years: B=−0.64, *p*=.048), shorter weekend sleep duration (≤6 hours: B=−0.83, *p*=.003), and not engaging in muscle-strengthening activities (B=−0.67, *p*=.004) were associated with lower health literacy among adult women.

**Conclusion:**

Interventions are needed to improve health literacy among adult Korean women, particularly among older women, those with lower education levels, and those with poorer perceived health. These interventions should also consider health behaviors such as muscle-strengthening activity and sleep duration. Further research is needed to examine the relationship between women’s health literacy and health behaviors, including exercise and sleep duration.

## Introduction

건강정보이해능력(health literacy)은 건강과 관련된 정보와 서비스에 접근하고, 이해하고, 평가하며, 활용할 수 있는 능력으로[1] 우리나라에서는 건강정보이해력, 건강문해력, 헬스리터러시 등으로 사용되기도 한다[2]. 건강정보이해능력은 일상생활과 사회적 상호작용을 통해 축적되는 지식과 역량으로, 단순히 건강 관련 정보를 읽고 건강 증진을 위한 행동을 따라 하는 것뿐만 아니라 건강 관련 정보를 탐색, 분석, 평가, 및 활용할 수 있는 비판적 사고능력을 포함한다[1].

건강정보이해능력은 건강과 건강 행동을 결정하는 중요한 요인이며[1,3] 건강 증진을 위한 중요한 개념으로[4], 질병 이환, 만성질환 관리, 건강 증진 행동, 의료서비스 이용 등 건강과 관련된 다양한 영역과 관련이 있다[5,6]. 건강정보이해능력이 높은 경우, 고혈압에 대한 지식수준이 높았고[7], 암 예방을 위한 행동[8]과 건강 증진 행동[9]을 더 많이 했으며, 의료서비스 이용은 낮은 것으로 보고되었다[5]. 건강정보이해능력이 낮은 경우 비만이나 장애 유병률이 높았고, 신체활동 빈도와 과일 및 채소 섭취 빈도는 낮았으며[4], 응급실 방문이나 외래 및 입원 빈도는 높은 것으로 보고되었다[4,6].

건강의 패러다임이 질병 치료 중심에서 예방 중심으로 전환되면서 건강관리를 위한 건강 정보의 중요성이 커지고 있으며[10] 건강 정보에 대한 관심 또한 높은 상태이다[6]. 이러한 경향에 맞추어 TV, 인터넷, 소셜미디어 등의 다양한 매체를 통해 수없이 많은 양의 건강 관련 정보들이 제공되고 있다[6,11]. 그러나 제공되고 있는 건강 관련 정보의 전문성과 신뢰성에 대한 문제가 지속적으로 제기되고 있는데[6] 건강정보이해능력이 부족한 경우 정확하지 않은 건강 정보를 구분하지 못할 수 있다[11]. 따라서 건강정보이해능력은 건강 정보 과잉 시대에 올바른 건강 정보에 근거한 건강관리를 위해 개인이 반드시 갖추어야 할 능력 중 하나라고 할 수 있다[10].

그러나 선행 연구에서 건강정보이해능력은 전반적으로 낮은 수준임을 보고하고 있다[3]. 경제적으로 발전한 유럽의 8개국을 대상으로 한 연구에서 많은 어린이, 청소년 및 성인의 건강정보이해능력이 낮은 수준인 것으로 보고되었으며[12], 국내 연구에서도 우리나라는 건강에 대한 관심은 높은 반면 건강정보이해능력은 건강 정보를 효율적으로 활용하고 의료서비스를 적절히 이용하기에는 충분하지 않은 수준으로 나타나 건강정보이해능력 증진을 위한 노력이 필요하다고 하였다[6,13]. 건강정보이해능력은 다양한 사회적 요인에 의해 형성되므로 건강정보이해능력의 증진 및 유지는 개인의 책임만은 아니다[1]. 따라서 건강정보이해능력 향상을 위한 국가적 차원의 중재 전략과 정책 수립이 필요하다[6].

건강정보이해능력 향상을 위한 중재는 건강 정보나 의료서비스의 접근 및 이용에 성별에 따른 차이가 존재하므로[14] 성별 맞춤형 중재를 제공하는 것이 필요하다[15,16]. 선행 연구에서는 남성에 비해 여성의 건강정보이해능력이 낮게 나타났음을 보고하였으나[5,13], 건강정보이해능력의 성별 차이에 대한 국내외 연구는 연구에 따라 서로 다른 결과를 보고하고 있어 특정 성별의 건강정보이해능력이 낮다고 해석하기는 어렵다[16]. 그러나 남성에 비해 여성은 외래 진료, 응급실 내원, 입원 등의 의료서비스 이용이 많은 것으로 보고되고 있다[5,17]. 우리나라의 경우 의료서비스 이용 증가로 건강보험 재정이 위협을 받고 있기 때문에 자신에게 필요한 의료서비스만 이용하는 의료서비스 이용의 합리성 확보가 필요하며 이를 위해서는 의료서비스 이용의 중요한 결정 요인인 건강정보이해능력 향상이 필요하다[5]. 따라서 남성에 비해 의료서비스 이용이 많은 여성의 건강정보이해능력 증진이 보다 시급한 과제라고 할 수 있다. 또한 여성은 가족을 돌보고 가족 구성원의 건강을 관리하는 건강 관리자 역할을 하기 때문에 여성의 건강정보이해능력은 가족의 건강 증진을 결정하는 요소로[18] 여성 자신뿐만 아니라 자녀의 건강 결과에도 영향을 미친다[19,20]. 따라서 건강정보이해능력 증진을 위한 전략 및 정책수립은 여성에게 우선적인 초점을 두는 것이 필요하다.

여성의 건강정보이해능력 증진을 위한 중재 전략 및 정책 수립을 위해서는 여성의 건강정보이해능력 수준을 파악하고 취약 계층을 파악하여 전략 및 정책 수립의 근거로 제시할 필요가 있다[2,9]. 건강정보이해능력과 관련하여 기존에 국내에서 실시된 대부분의 선행 연구는 남녀 모두를 대상으로 하여 건강정보이해능력의 성별에 따른 차이는 파악하였으나 남성과 여성을 구분하여 성별에 따른 관련 요인을 파악한 연구는 거의 없어, 여성의 건강정보이해능력과 관련된 요인에 대한 연구는 매우 부족한 실정이다. 또한 대부분의 연구는 일부 집단을 대상으로 건강정보이해능력 수준 및 관련 요인을 파악하고 있으며 국가적 차원에서 대표성 있는 표본을 대상으로 한 연구는 부족한 편이다[6,13]. 이에 본 연구에서는 국가 단위의 조사인 국민건강영양조사 자료를 이용하여 19세 이상 성인 여성 전체를 대상으로 건강정보이해능력 수준을 파악하고 관련 요인을 파악하고자 하며 이를 통해 여성의 건강정보이해능력 증진을 위한 전략 및 정책 수립을 위한 근거 자료를 제공하고자 한다.

본 연구의 목적은 한국 성인 여성의 건강정보이해능력 수준 및 관련 요인을 파악하기 위함이며 본 연구의 구체적인 목적은 다음과 같다.

1) 성인 여성의 건강정보이해능력 수준을 파악한다.

2) 성인 여성의 일반적 특성 및 건강 관련 특성에 따른 건강정보이해능력의 차이를 파악한다.

3) 성인 여성의 건강정보이해능력과 관련된 요인을 파악한다.

## Methods

**Ethics statement:** This study was secondary analysis using the Korea National Health and Nutrition Examination Survey data and the data were received in an anonymized format. As such, Institutional Review Board approval was not required but the study adhered to principles of the Declaration of Helsinki.

### 연구 설계

본 연구는 성인 여성의 건강정보이해능력 및 관련 요인을 파악하기 위해 2023년에 질병관리청에서 실시한 국민건강영양조사 제9기 2차년도 자료를 이차 분석한 상관성 연구이다. 본 연구의 기술은 STROBE 가이드라인을 준수하여 작성하였다(https://www.strobe-statement.org/).

### 연구 대상

**Figure 1.**
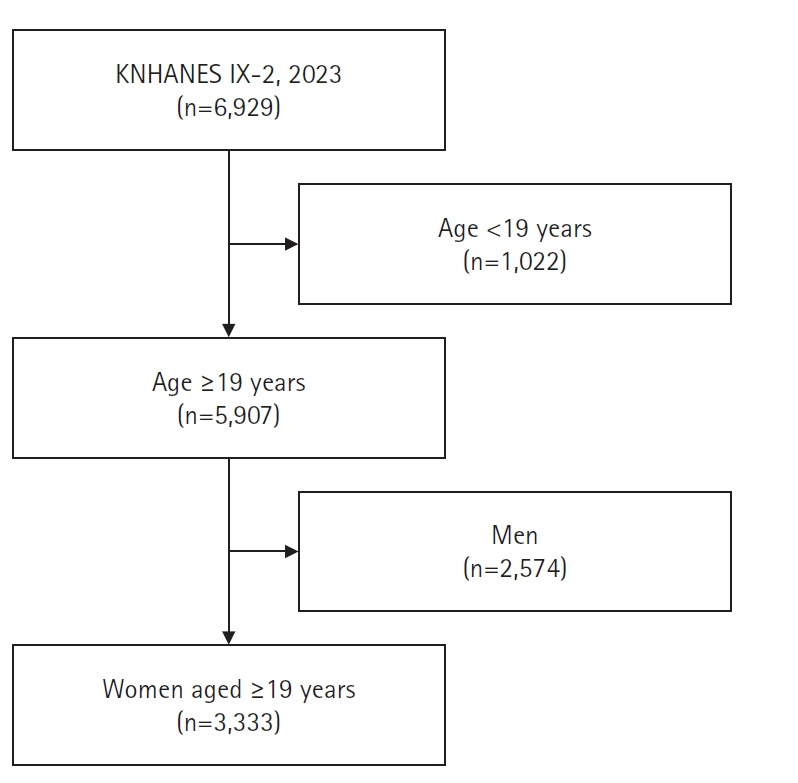
Flowchart of the study population. KNHANES: Korea National Health and Nutritional Examination Survey.

본 연구는 질병관리청에서 2023년 1월에서 12월까지 실시한 국민건강영양조사 제9기 2차년도에 참여한 19세 이상 성인 여성 전체를 대상으로 하였다. 국민건강영양조사 제9기 2차년도 참여자는 총 6,929명으로, 19세 이상의 성인은 5,907명이었으며, 성인 여성은 3,333명이었다(Figure 1).

### 연구 변수

#### 건강정보이해능력

본 연구에서 건강정보이해능력은 국민건강영양조사의 건강정보이해능력 자료를 사용하였다. 국민건강영양조사에서 건강정보이해능력은 총 10문항으로 구성되어 있다. 각 문항은 4점 척도로 ‘전혀 그렇지 않다’ 1점, ‘그렇지 않다’ 2점, ‘그렇다’ 3점, ‘매우 그렇다’ 4점으로 측정하며, 총점 범위는 10–40점으로 점수가 높을수록 건강정보이해능력이 높음을 의미한다. 건강정보이해능력 수준은 선행 연구[21]를 참고하여, 건강정보이해능력 총점을 기준으로 32점 이상은 ‘우수’, 28점에서 31점은 ‘중간’, 27점 이하는 ‘부족’으로 분류하였다.

#### 일반적 특성

본 연구에서 일반적 특성은 국민건강영양조사의 기본조사 및 건강설문조사 중 거주 지역, 나이, 소득 4분위수(가구), 교육 수준 재분류 코드, 결혼 여부, 직업 재분류 및 실업/비경제활동 상태 코드 및 가구 세대 구성 코드 자료를 사용하였다.

지역은 거주 지역 자료를 사용하여 동은 ‘도시’, 읍∙면은 ‘시골’로 분류하였고, 나이는 만 나이를 사용하였다. 나이는 중년과 노년을 구분하여 '19–44세', '45–64세(중년)', '65세 이상(노년)'으로 분류하였다. 소득은 개방형으로 작성된 가구 총소득을 월평균 가구 균등화 소득에 따라 4개 군으로 등분하여 '하', '중하', '중상', '상'으로 분류한 소득 4분위수(가구) 자료를 사용하였다. 교육 수준은 '초졸 이하', '중졸', '고졸', '대졸 이상'으로 분류한 교육 수준 재분류 코드 자료를 사용하였고, 결혼 상태는 ‘미혼’과 ‘기혼’으로 분류한 결혼 여부 자료를 사용하였다. 직업은 6개의 직업군과 무직으로 분류한 직업 재분류 및 실업/비경제활동 코드 자료를 사용하여 6개 직업군은 ‘예(직업 있음)’, 무직은 ‘아니오(직업 없음)’으로 분류하였다. 세대 유형은 7개의 가구 형태로 분류한 가구 세대 구성 코드 자료를 사용하여 1세대-1인 가구는 ‘일인 가구’, 나머지는 ‘다인 가구’로 분류하였다.

#### 건강 관련 특성

본 연구에서 건강 관련 특성은 국민건강영양조사의 건강설문조사 자료 중 월경 여부, 주관적 건강 인지, 평소 스트레스 인지 정도, 2주 이상 연속 우울감 여부, 1년간 자살 생각 여부, 월간 음주율, 현재 흡연율, 1주일간 걷기 일수 및 걷기 지속 시간, 유산소 신체활동 실천율, 1주일간 근력운동 일수, 주중(또는 일하는 날) 하루 평균 수면시간 및 주말(또는 일하지 않는 날, 일하지 않는 전날) 하루 평균 수면시간 자료를 활용하였다.

폐경 여부는 월경 여부 자료를 사용하여 자연 폐경과 인공 폐경은 ‘예’, 나머지는 ‘아니오’로 분류하였다. 건강인지는 주관적 건강인지 자료를 사용하여 매우 좋음과 좋음은 ‘좋음’, 보통은 ‘보통’, 나쁨과 매우 나쁨은 ‘나쁨’으로 분류하였다. 스트레스 인지 여부는 평소 스트레스 인지 정도 자료를 사용하여 ‘대단히 많이 느낀다’와 ‘많이 느끼는 편이다’는 ‘예’, ‘조금 느끼는 편이다’와 ‘거의 느끼지 않는다’는 ‘아니오’로 분류하였다. 우울감 여부는 2주 이상 연속 우울감 여부 자료를 사용하여 ‘예’와 ‘아니오’로 분류하였고, 자살 생각 여부는 1년간 자살 생각 여부 자료를 사용하여 ‘예’와 ‘아니오’로 분류하였다. 음주 여부는 월간 음주율 자료를 사용하여 최근 1년간 월 1잔 이상 음주는 ‘예’, 평생 비음주와 최근 1년간 월 1잔 미만 음주는 ‘아니오’로 분류하였고, 흡연 여부는 현재 흡연율 자료를 사용하여 현재 흡연은 ‘예’, 과거 흡연과 비흡연은 ‘아니오’로 분류하였다. 걷기 여부는 1주일간 걷기 일수와 걷기 지속 시간 자료를 사용하여, 걷기 실천율 정의[22]를 참고하여 1일 총 30분 이상 주 5일 이상 실천한 경우는 ‘예’, 나머지는 ‘아니오’로 분류하였다. 신체활동 여부는 일주일에 중강도 신체활동을 2시간 30분 이상 또는 고강도 신체활동을 1시간 15분 이상 또는 중강도와 고강도 신체활동을 섞어서 각 활동에 상당하는 시간을 실천한 경우는 ‘예’ 나머지는 ‘아니오’로 분류한 유산소 신체활동 실천율 자료를 사용하였다. 근력운동 여부는 1주일간 근력운동 일수 자료를 사용하여 세계보건기구의 가이드라인[23]을 참고하여 일주일에 2일 이상은 ‘예’, 일주일에 2일 미만은 ‘아니오’로 분류하였다. 주중 및 주말 수면시간은 주중(또는 일하는 날)과 주말(또는 일하지 않는 날, 일하지 않는 전날) 하루 평균 수면시간 자료를 사용하고 선행 연구[24]를 참고해 ‘6시간 이하’, ‘7–8시간’, ‘9시간 이상’으로 분류하였다.

### 자료수집 및 분석

본 연구의 자료는 질병관리청의 국민건강영양조사 누리집에서 통계자료 이용자 준수사항 이행 서약서 작성 및 사용자 정보 등록을 마친 후 2023년 원시자료를 다운받아 사용하였다. 다운받은 자료는 IBM SPSS ver. 20.1 (IBM Corp., Armonk, NY, USA)을 이용하여 분석하였으며, 층, 집락, 가중치를 고려한 복합 표본 분석법을 사용하였다. 성인 여성의 건강정보이해능력 총점 및 문항별 점수는 복합 표본 기술 통계로 분석하였고, 건강정보이해능력 수준, 일반적 특성 및 건강관련 특성은 복합 표본 빈도 분석을 실시하였다. 성인 여성의 일반적 특성 및 건강관련 특성에 따른 건강정보이해능력의 차이와 성인 여성의 건강정보이해능력 관련 요인은 복합 표본 일반 선형 모형으로 분석하였다.

## Results

### 성인 여성의 일반적 특성 및 건강관련 특성

성인 여성은 대부분 도시(84.6%)에 거주하고 있었으며, 나이는 19–44세(38.4%)가 가장 많았다. 소득은 상(29.5%)인 여성이 가장 많았고, 교육 수준은 대졸 이상(42.0%)이 가장 많았다. 결혼 상태는 기혼(79.1%)이 더 많았고, 직업이 있는 여성(56.7%)이 더 많았으며, 세대 유형은 다인 가구(83.7%)가 더 많았다(Table 1).

폐경을 하지 않은 여성(51.0%)이 폐경 여성(49.0%)보다 조금 더 많았고, 자신의 건강을 보통으로 인지하는 여성(48.2%)이 가장 많았다. 스트레스를 인지하지 않는 여성(72.9%)이 더 많았고, 우울감이 없는 여성(86.3%)이 더 많았으며, 자살 생각 경험이 없는 여성(94.6%)이 더 많았다. 음주를 하지 않는 여성(56.6%)이 더 많았고, 흡연을 하지 않는 여성(94.9%)이 더 많았다. 걷기를 실천하는 여성(53.3%)이 더 많았으며, 신체활동(53.4%)과 근력운동(81.7%)을 실천하지 않는 여성이 더 많았다. 주중 수면시간(49.1%)과 주말 수면시간(49.7%)은 7–8시간이 가장 많았다(Table 2).

### 성인 여성의 건강정보이해능력

성인 여성의 건강정보이해능력은 평균 30.25점으로 중간 수준인 것으로 나타났다. 문항별 점수는 7번 문항(의사나 약사가 설명해 주는 약 먹는 방법 이해)이 4점 만점에 3.31점으로 가장 높았고, 9번 문항(인터넷이나 미디어에서 얻은 건강 정보가 믿을 만한 것인지 판단)이 4점 만점에 2.89점으로 가장 낮은 것으로 나타났다. 건강정보이해능력 수준은 32.2%가 ‘우수’에 해당했으며, 67.8%는 ‘중간’ 또는 ‘부족’에 해당하는 것으로 나타났다(Table 3).

### 성인 여성의 일반적 특성에 따른 건강정보이해능력 차이

성인 여성의 건강정보이해능력은 지역, 나이, 소득, 교육 수준, 결혼 상태, 직업, 및 세대 유형에 따라 차이가 있었다. 성인 여성의 건강정보이해능력은 도시에 거주하는 여성에 비해 시골에 거주하는 여성에서 낮게 나타났고(t=15.03, *p*<.001), 나이가 많을수록 낮게 나타났다(F=126.24, *p*<.001). 소득이 낮을수록 건강정보이해능력이 낮았고(F=35.31, *p*<.001), 교육 수준이 낮을수록 건강정보이해능력이 낮게 나타났다(F=139.88, *p*<.001). 미혼 여성에 비해 기혼 여성의 건강정보이해능력이 낮았고(t=36.13, *p*<.001), 직업이 있는 여성에 비해 직업이 없는 여성의 건강정보이해능력이 낮았으며(t=17.30, *p*<.001), 다인 가구 여성에 비해 일인 가구 여성의 건강정보이해능력이 낮게 나타났다(t=52.78, *p*<.001) (Table 1).

### 성인 여성의 건강관련 특성에 따른 건강정보이해능력 차이

성인 여성의 건강정보이해능력은 폐경, 건강인지, 우울감, 자살 생각, 음주, 신체활동, 근력운동, 주중 수면시간 및 주말 수면시간에 따라 차이가 있었다. 폐경을 하지 않은 여성에 비해 폐경 여성에서 건강정보이해능력이 낮게 나타났고(t=187.81, *p*<.001), 자신의 건강을 나쁘다고 인지하는 여성에서 건강정보이해능력이 가장 낮은 것으로 나타났다(F=44.95, *p*<.001). 우울감이 없는 여성에 비해 우울감이 있는 여성에서 건강정보이해능력이 낮게 나타났고(t=7.49, *p*=.007), 자살 생각 경험이 없는 여성에 비해 자살 생각 경험이 있는 여성에서 건강정보이해능력이 낮게 나타났다(t=6.98, *p*=.009). 음주 여성에 비해 비음주 여성에서 건강정보이해능력이 낮게 나타났고(t=54.17, *p*<.001), 신체활동을 하지 않는 여성(t=39.46, *p*<.001)과 근력운동을 실천하지 않는 여성에서(t=30.17, *p*<.001) 건강정보이해능력이 낮게 나타났다. 주중 수면시간은 9시간 이상인 여성에서(F=8.03, *p*<.001), 주말 수면시간은 6시간 이하인 여성에서(F=36.81, *p*<.001) 건강정보이해능력이 가장 낮게 나타났다(Table 2).

### 성인 여성의 건강정보이해능력 관련 요인

성인 여성의 건강정보이해능력은 나이, 교육 수준, 건강인지, 근력운동, 주중 수면시간 및 주말 수면시간과 관련이 있었으며 설명력은 21%이었다. 19–44세 여성에 비해 45–64세 여성(B=–0.64, *p*=.048)과 65세 이상 여성(B=–1.43, *p*=.001)의 건강정보이해능력이 낮았고, 대졸 이상인 여성에 비해 고졸 여성(B=–0.84, *p*<.001), 중졸 여성(B=–2.17, *p*<.001), 초졸 이하 여성(B=–4.31, *p*<.001)의 건강정보이해능력이 낮게 나타났다. 자신의 건강을 좋다고 인지하는 여성에 비해 보통으로 인지하는 여성(B=–0.82, *p*<.001)과 나쁘다고 인지하는 여성(B=–1.49, *p*<.001)의 건강정보이해능력이 낮았고, 근력운동을 실천하는 여성에 비해 실천하지 않는 여성의 건강정보이해능력이 낮게 나타났다(B=–0.67, *p*=.004). 주중 수면시간이 7–8시간인 여성에 비해 9시간 이상인 여성의 건강정보이해능력이 낮았고(B=–1.54, *p*=.003), 주말 수면시간이 7–8시간인 여성에 비해 6시간 이하인 여성의 건강정보이해능력이 낮게 나타났다(B=–0.83, *p*=.003) (Table 4).

## Discussion

본 연구는 국가 단위 조사인 국민건강영양조사 자료를 이용하여 성인 여성의 건강정보이해능력을 파악하고 건강정보이해능력과 관련된 요인을 파악하기 위해 수행하였다.

본 연구에서 성인 여성의 건강정보이해능력은 평균 30.25점으로 ‘중간’ 수준인 것으로 나타났다. 또한 성인 여성의 32.2%는 ‘우수’ 수준의 건강정보이해능력을 갖추고 있는 것으로 나타났는데, 이는 건강정보이해능력 수준에 대한 선행 연구에서 22.7%의 여성이 우수한 수준이었음을 보고한 결과[25]에 비해 높은 수준이며, 성인의 50.6%가 우수한 수준이었음을 보고한 결과[13]에 비해 낮은 수준이다. 건강정보이해능력은 연구에 따라 측정 도구를 다르게 사용하고 있으며, 건강정보이해능력 수준 구분도 연구에 따라 다른 기준을 적용하고 있어 직접적인 비교에는 무리가 있다. 선행 연구에서 우리나라 국민의 건강정보이해능력은 낮은 수준으로 보고하였고[6], 정부에서도 제5차 국민건강증진종합계획(Health Plan 2030)에 건강정보이해력 제고를 중점 과제 중 하나로 선정하면서 건강정보이해력 증진을 위한 정책적 접근을 시작하였다[5]. 건강정보이해능력 증진을 위해서는 건강정보이해능력 수준을 지속적으로 모니터링하고 교육기관, 의료기관, 정부 등 다양한 기관의 협력을 통해 다각적인 전략 수립이 필요하다[6]. 따라서 여성의 건강정보이해능력 수준을 지속적으로 모니터링하고 관련 요인을 파악하여 취약 계층을 위한 다양한 전략 수립이 필요하다.

또한 본 연구에서 건강정보이해능력 문항별 점수는 ‘인터넷이나 미디어에서 얻은 건강 정보가 믿을 만한 것인지 판단’하는 문항에서 가장 낮게 나타났으며, ‘인터넷이나 미디어에서 얻은 건강 정보를 건강과 관련한 행동이나 의사결정에 활용’ 문항에서 그다음으로 낮게 나타났다. 여성은 남성에 비해 건강 관련 TV 프로그램을 더 많이 보며[26], 건강 정보를 수집하기 위한 인터넷 활용을 더 많이 하는 것으로 보고되나[27] 매체를 통해 얻은 정보를 판단하고 활용하는 능력은 부족하다는 것을 보여준다. 건강 정보 과잉 시대라고 할 만큼 다양한 매체를 통해 다양한 건강 정보가 제공되고 있는 반면, 제공되는 정보들의 전문성과 신뢰성에 대한 문제가 지속적으로 제기되고 있다[6,10]. 건강정보이해능력 증진을 위해서는 신뢰할 수 있는 정보에 접근할 수 있도록 사회적 환경을 조성하는 것이 필요하며 이를 위한 전략에는 건강 정보를 접하고 활용하는 정보 환경과 인터넷, 방송 등의 매체에 대한 규제도 포함된다[1]. 따라서 여성의 건강정보이해능력 증진을 위해서는 믿을 수 있는 전문적인 건강 정보 제공뿐 아니라 활용 가능한 형태의 정보 제공 및 정보의 판단과 활용에 대한 교육이 필요하며, 건강 정보를 제공하는 매체에 대한 규제를 병행하는 것이 필요하다.

본 연구에서 성인 여성의 건강정보이해능력은 나이가 많을수록 낮은 것으로 나타나 선행 연구[25,28] 결과와 일치하였다. 그러나 젊은 연령대에서 건강정보이해능력이 낮게 나타났음을 보고한 연구[29] 및 건강정보이해능력과 나이는 관련이 없음을 보고한 연구[30]와는 차이가 있었는데, 이는 노인의 교육 수준이 높은 국가적인 특성[29]과 65세 미만의 특정 연령대에 집중된 대상자의 특성[30] 때문이라고 할 수 있다. 연령이 증가하면 인지 및 이해 능력과 기억 능력이 감소하기 때문에 건강정보이해능력도 감소하며 최근에는 건강 정보를 제공하는 매체가 디지털화되고 있어 노인의 건강정보이해능력은 더욱 낮아질 수 있다[5]. 그러나 노인은 다른 연령대에 비해 건강에 대한 관심도가 높고 건강 행위를 실천하고자 하는 의지가 강하기 때문에 적절한 교육을 제공하는 경우 건강정보이해능력을 높일 수 있다[5,31]. 따라서 여성 노인을 대상으로 한 교육을 강화하는 것이 필요하며, 여성은 남성에 비해 건강 관련 TV 프로그램을 많이 보는 경향이 있으므로[26] TV를 통한 교육프로그램을 활성화하는 것을 고려할 수 있다. 또한 여성은 남성에 비해 디지털 기술 사용 능력이 낮으므로[16] 디지털 관련 교육을 강화하고 여성 노인들도 쉽게 이용할 수 있는 온라인 교육 프로그램을 개발하는 것이 필요하다고 할 것이다.

본 연구에서 성인 여성의 건강정보이해능력은 교육 수준이 낮을수록 낮게 나타나, 선행 연구[18,25] 결과와 일치하였다. 선행 연구에서 교육 수준은 건강정보이해능력의 주요한 결정 요인으로 교육 수준이 높을수록 건강정보이해능력이 높은 것으로 보고하고 있다[29,30]. 건강정보이해능력은 건강 정보를 이해하고 평가하여 적용하는 능력이므로[1] 교육 수준이 높으면 건강정보이해능력이 높을 수 있다. 그러나 교육 수준은 높지만 부적절한 건강 정보를 가지고 있는 경우도 있고 건강정보이해능력이 낮은 경우도 있기 때문에 최종 학력만으로 건강정보이해능력을 판단하는 것은 적절하지 않을 수 있다[18]. 따라서 성인 여성의 건강정보이해능력 향상을 위한 중재는 교육 수준이 낮은 여성에게 우선적으로 제공하는 것이 필요하며, 교육 수준이 높은 여성인 경우에도 건강정보이해능력 수준을 파악하고 잘못된 정보의 수정 및 건강정보이해능력 수준별 중재를 제공하는 것이 필요하다고 할 것이다.

본 연구에서 성인 여성의 건강정보이해능력은 자신의 건강을 좋음으로 인지한 여성에 비해 보통 또는 나쁨으로 인지한 여성에서 낮은 것으로 나타나 선행 연구[29,32]의 결과와 일치하였다. 그러나 건강 인지와 건강정보이해능력은 관계가 없음을 보고한 선행 연구[30,33]와는 차이가 있는데, 이는 선행 연구에서는 좋음과 나쁨의 두 가지로 구분하거나[30] 보통∙좋음과 나쁨으로 구분하고 있어[33] 건강 인지에 대한 구분이 다르기 때문일 수 있다. 건강정보이해능력이 낮은 경우 자신의 건강 상태를 정확하게 판단하는 능력이 부족하고[31], 의료서비스 이용을 많이 하며[5], 불필요한 의료서비스를 이용하는 경향이 있는 것으로 보고되고 있다[34,35]. 이를 고려하면 본 연구에서 자신의 건강을 보통 또는 나쁨으로 인지한 여성에서 건강정보이해능력이 낮게 나타난 결과는 건강정보이해능력이 낮은 여성이 자신의 건강 상태를 부정적으로 평가했기 때문일 수 있다. 따라서 성인 여성의 건강정보이해능력 향상을 위한 중재는 자신의 건강 상태에 대해 부정적으로 인지하는 여성에게 우선 적용하는 것이 필요하다. 자신의 건강 상태에 대해 긍정적으로 인지하지 못하는 경우 이에 대한 이유나 근거를 파악하여 건강 상태에 따른 필요한 중재를 제공하고, 잘못된 부분이 있는 경우 자신의 건강 상태에 대한 올바른 판단을 위한 교육적 중재를 제공하는 것이 필요하다.

본 연구에서 성인 여성의 건강정보이해능력은 주 2일 이상의 근력운동을 실천하는 경우에 비해 근력운동을 실천하지 않는 경우에서 낮게 나타났다. 건강정보이해능력과 근력운동 실천의 관계에 대한 선행 연구가 없어 직접적인 비교에는 무리가 있다. 그러나 건강정보이해능력과 신체활동의 관계에 대한 체계적 고찰에서 대부분의 연구가 건강정보이해능력과 신체활동이 유의미한 양의 상관관계가 있음을 제시하였다고 보고하였다[36]. 건강정보이해능력과 운동, 금연, 절주 등의 건강 증진 행위와의 관계에 대한 국내외 연구는 건강정보이해능력이 높을수록 건강 증진 행위를 많이 하는 것으로 보고하고 있다[11,28]. 그러나 건강 증진 행위와 건강정보이해능력은 관계가 없음을 보고하기도 하였는데[29,30], 이는 건강정보이해능력이 건강 행동에 대한 중요한 정보는 제공하지만 건강 행동에 영향을 미치는 유일한 요인은 아니기 때문이라고 하였다[29]. 본 연구에서도 근력운동 실천은 건강정보이해능력과 관련이 있었으나, 걷기 및 신체활동 실천은 건강정보이해능력과 관련이 없는 것으로 나타났다. 따라서 건강 증진 행동과 건강정보이해능력의 관계에 대해서는 추후 지속적인 연구가 필요하다. 또한 본 연구에서 성인 여성의 근력운동 실천율은 18.3%로 낮게 나타났고, 여성은 남성에 비해 근력운동 실천율이 낮아 여성의 근력운동 실천을 높이기 위한 전략이 필요함을 고려하면[37], 여성의 근력운동 실천과 건강정보이해능력의 관계에 대한 지속적인 연구를 통한 근거 자료 축적이 필요하다.

본 연구에서 성인 여성의 건강정보이해능력은 주중 수면시간이 9시간 이상이거나 주말 수면시간이 6시간 이하인 여성에서 낮은 것으로 나타났다. 이는 6시간 미만의 수면과 낮은 수준의 건강정보이해능력이 관계가 있음을 보고한 선행 연구[38]와 부분적으로 일치하는 결과이며, 높은 수준의 건강정보이해능력과 적절한 수면이 관계가 있음을 보고한 선행 연구[39]와 같은 맥락에서 이해할 수 있다. 그러나 수면과 건강정보이해능력의 관계에 대한 연구는 부족하며, 수면시간 관련 연구는 거의 없는 실정이다. 따라서 건강 증진 행동과 건강정보이해능력의 관계에 대한 연구에서 수면시간도 주요한 건강 증진 행동으로 포함할 필요가 있으며, 반복 연구를 통한 근거 축적이 필요하다. 또한 본 연구에서 주중 수면시간과 주말 수면시간에 따른 건강정보이해능력이 서로 다른 양상을 보였으므로, 추후 연구에서도 주중 수면시간과 주말 수면시간을 구분하여 파악하는 것이 필요하다.

본 연구는 다음과 같은 제한점을 가진다. 첫째, 본 연구에서는 단면적 연구 설계를 사용했기 때문에 건강정보이해능력과 건강 행동의 인과관계를 규명하는 데에는 한계가 있다. 따라서 추후 연구에서는 건강정보이해능력과 건강 행동의 인과관계 규명을 위한 전향적 연구가 필요하다. 둘째, 본 연구에서는 건강인지 등 주관적 건강 상태를 반영했으며 질환 등의 객관적 건강 상태를 반영하지 못했다. 따라서 추후 연구에서는 주관적 및 객관적 건강 상태와 건강정보이해능력의 관련성을 비교하는 연구가 필요하다. 마지막으로, 본 연구에서 사용한 건강정보이해능력 측정 도구는 선행 연구에서 사용한 도구와 차이가 있어 본 연구 결과와 선행 연구 결과의 직접적인 비교에는 무리가 있을 수 있다. 따라서 추후 계속적인 반복 연구를 통해 근거를 축적하는 것이 필요하다.

이러한 제한점에도 불구하고 본 연구는 다음과 같은 의의가 있다. 첫째, 본 연구는 국가적 대표성을 가진 자료인 2023년도 국민건강영양조사 데이터를 활용하여 성인 여성의 건강정보이해능력 및 관련 요인을 분석하였기 때문에 연구 결과의 신뢰성과 일반화 가능성을 높였다고 볼 수 있다. 둘째, 본 연구에서는 여성만을 대상으로 하여 건강정보이해능력 및 관련 요인을 파악하였기 때문에 건강정보이해능력 증진을 위한 여성 맞춤형 중재 전략을 수립할 수 있는 근거를 제공하였다. 마지막으로, 본 연구에서는 여성의 건강정보이해능력 관련 요인을 파악함에 있어 인구사회학적 특성뿐 아니라 건강 관련 특성을 포함하여 여성의 건강정보이해능력 관련 요인에 대한 종합적인 분석을 시도하였다는 점에서 의의가 있다.

결론적으로 본 연구는 성인 여성의 건강정보이해능력 향상이 필요하며 나이, 교육 수준, 건강인지, 근력운동, 및 수면시간을 고려한 중재 제공이 필요함을 제시하였다. 따라서 본 연구 결과는 성인 여성의 건강정보이해능력 증진 프로그램 개발을 위한 근거로 활용할 수 있을 것이다. 또한 본 연구에서 근력운동, 수면시간 등의 건강 증진 행동과 건강정보이해능력의 관련성을 제시하였으므로 건강 증진 행동과 건강정보이해능력의 관계에 대한 지속적인 연구를 통해 근거를 축적하는 것이 필요하다고 할 것이다.

## Figures and Tables

**Table 1.** Differences in health literacy according to general characteristics (N=3,333)

Characteristics	Categories	n^†^	%^‡^ (SE)	Health literacy	
				Mean (SE)	t or F (*p*)
Region	City	2,687	84.6 (2.5)	30.57 (0.15)	15.03 (<.001)
	Rural	646	15.4 (2.5)	28.44 (0.52)	
Age (year)	19–44	1,007	38.4 (1.2)	31.93 (0.16)	126.24 (<.001)
	45–64	1,302	37.8 (1.0)	30.66 (0.16)	
	≥65	1,024	23.9 (1.1)	26.70 (0.30)	
Income	Low	725	18.8 (1.0)	27.19 (0.40)	35.31 (<.001)
	Middle-low	821	24.0 (1.0)	30.21 (0.20)	
	Middle-high	865	27.7 (1.1)	30.81 (0.21)	
	High	906	29.5 (1.5)	31.58 (0.18)	
Education	≤Elementary school	639	15.6 (1.0)	25.28 (0.36)	139.88 (<.001)
	Middle school	337	8.5 (0.5)	28.58 (0.29)	
	High school	1,050	34.0 (1.1)	30.71 (0.15)	
	≥University	1,215	42.0 (1.5)	32.06 (0.14)	
Marital status	Unmarried	505	20.9 (1.1)	31.52 (0.24)	36.13 (<.001)
	Married	2,828	79.1 (1.1)	29.91 (0.16)	
Occupation	No	1,381	43.3 (1.1)	29.80 (0.23)	17.30 (<.001)
	Yes	1,685	56.7 (1.1)	30.75 (0.14)	
Household type	Single-person	617	16.3 (1.0)	28.09 (0.36)	52.78 (<.001)
	Multi-person	2,716	83.7 (1.0)	30.65 (0.14)	

† Unweighted and valid frequency;

‡ weighted percentile.

**Table 2.** Differences in health literacy according to health-related characteristics (N=3,333)

Characteristics	Categories	n^†^	%^‡^ (SE)	Health literacy	
				Mean (SE)	t or F (p)
Menopause	No	1,270	51.0 (1.3)	31.85 (0.15)	187.81
	Yes	1,787	49.0 (1.3)	28.76 (0.18)	(<.001)
Perceived health	Poor	658	21.0 (0.9)	28.64 (0.27)	44.95 (<.001)
	Ordinary	1,520	48.2 (1.1)	30.28 (0.15)	
	Good	900	30.8 (1.1)	31.59 (0.20)	
Perceived stress	No	2,416	72.9 (0.8)	30.29 (0.16)	0.36 (.547)
	Yes	832	27.1 (0.8)	30.15 (0.22)	
Depressive symptoms	No	2,819	86.3 (0.7)	30.36 (0.15)	7.49 (.007)
	Yes	430	13.7 (0.7)	29.54 (0.30)	
Suicidal ideation	No	3,090	94.6 (0.5)	30.31 (0.15)	6.98 (.009)
	Yes	158	5.4 (0.5)	29.17 (0.41)	
Current drinking	No	1,951	56.6 (1.2)	29.56 (0.18)	54.17 (<.001)
	Yes	1,300	43.4 (1.2)	31.14 (0.17)	
Current smoking	No	3,104	94.9 (0.6)	30.26 (0.15)	0.30 (.585)
	Yes	144	5.1 (0.6)	29.98 (0.52)	
Walking	No	1,228	46.7 (1.1)	30.47 (0.20)	0.83 (.363)
	Yes	1,406	53.3 (1.1)	30.66 (0.15)	
Physical activity	No	1,723	53.4 (1.2)	29.73 (0.19)	39.46 (<.001)
	Yes	1,338	46.6 (1.2)	31.07 (0.15)	
Muscle strengthening	No	2,516	81.7 (0.9)	30.09 (0.15)	30.17 (<.001)
activity	Yes	555	18.3 (0.9)	31.42 (0.22)	
Weekday sleep duration (hour/day)	≤6	1,542	46.4 (1.0)	30.02 (0.18)	8.03 (<.001)
	7–8	1,567	49.1 (1.0)	30.64 (0.18)	
	≥9	131	4.4 (0.4)	29.93 (0.55)	
Weekend sleep duration (hour/day)	≤6	1,038	29.0 (0.9)	28.98 (0.22)	36.81 (<.001)
	7–8	1,614	49.7 (0.9)	30.59 (0.18)	
	≥9	588	21.4 (1.0)	31.29 (0.24)	

† Unweighted and valid frequency;

‡ weighted percentile.

**Table 3.** Health literacy of respondents (N=3,333)

Variables	Categories	Range or n^†^	Value
Health literacy items	7. Understand instructions on how to take a prescribed medicine.	1–4	3.31 (0.01)
	5. Understand what your doctor says to you during treatment.	1–4	3.22 (0.01)
	8. Understand patient education materials.	1–4	3.15 (0.02)
	3. Understand your health risk based on behaviors such as drinking, smoking, lack of exercise.	1–4	3.02 (0.02)
	4. Recognize which everyday behaviors are related to your health.	1–4	3.02 (0.02)
	6. Know what to do first in a medical emergency.	1–4	2.97 (0.01)
	2. Understand your mental health risk based on factors such as stress and depression.	1–4	2.95 (0.02)
	1. Determine which vaccinations you may need.	1–4	2.93 (0.02)
	10. Use information on the internet or in the media for health-related decision-making and health behaviors.	1–4	2.91 (0.02)
	9. Judge whether health-related information on the internet or in the media is reliable.	1–4	2.89 (0.02)
	Sum	10–40	30.25 (0.15)
Health literacy level	Excellent (≥32)	957	32.2% (1.00)
	Ordinary (28–31)	1,560	47.9% (1.00)
	Insufficient (≤27)	718	19.9% (1.00)

Values are presented as mean (SE) or weighted percentile (SE).

† Unweighted and valid frequency.

**Table 4.** Factors associated with health literacy

Variables	Categories	B	SE	95% CI	t	*p*
Residence^†^	City	0.56	0.35	–0.12 to 1.24	1.61	.107
Age (year)^†^	≥65	–1.43	0.44	–2.29 to –0.56	–4.95	.001
	45–64	–0.64	0.32	–1.27 to –0.01	–2.54	.048
Income^†^	Low	–0.46	0.37	–1.18 to 0.27	–1.23	.219
	Middle-low	–0.04	0.26	–0.51 to 0.50	–0.02	.987
	Middle-high	–0.35	0.27	–0.87 to 0.17	–1.33	.187
Education^†^	≤Elementary school	–4.31	0.40	–5.10 to –3.52	–11.19	<.001
	Middle school	–2.17	0.41	–2.98 to –1.36	–5.64	<.001
	High school	–0.84	0.20	–1.24 to –0.44	–3.83	<.001
Marital status^†^	Unmarried	–0.52	0.27	–1.04 to 0.02	–1.92	.057
Occupation^†^	No	0.18	0.21	–0.23 to 0.59	0.86	.392
Household type^†^	Single-person	–0.37	0.30	–0.97 to 0.24	–1.20	.231
Menopause	Yes	–0.24	0.32	–0.87 to 0.40	–0.73	.468
Perceived health^†^	Bad	–1.49	0.28	–2.03 to –0.94	–5.39	<.001
	Ordinary	–0.82	0.21	–1.24 to –0.41	–3.93	<.001
Depressive symptom^†^	Yes	–0.18	0.30	–0.77 to 0.41	–0.61	.540
Suicidal ideation^†^	Yes	–0.66	0.46	–1.58 to 0.25	–1.43	.154
Alcohol drinking^†^	No	–0.15	0.19	–0.53 to 0.23	–0.78	.436
Physical activity^†^	No	–0.20	0.18	–0.56 to 0.15	–1.13	.261
Muscle activity strengthening^†^	No	–0.67	0.23	–1.12 to –0.21	–2.90	.004
Weekday sleep duration^†^ (hour/day)	≤6	0.38	0.24	–0.10 to 0.86	1.56	.120
	≥9	–1.54	0.52	–2.56 to –0.52	–2.98	.003
Weekend sleep duration^†^ (hour/day)	≤6	–0.83	0.28	–1.38 to –0.28	–2.98	.003
	≥9	0.34	0.30	–0.25 to 0.93	1.13	.260
R^2^=.21, F=21.86, *p*<.001						

CI: Confidence interval.

† The reference categories for variables were residence (rural), age (19–44 years), income (high), education (≥university), marital status (married), occupation (yes), household type (multi-person), menopause (no), perceived health (good), depressive symptom (no), suicidal ideation (no), alcohol drinking (yes), physical activity (yes), muscle strengthening activity (yes), weekday sleep time (7–8 hours/day), weekend sleep time (7–8 hours/day).
